# Femtosecond Laser-Fabricated Photonic Chips for Optical Communications: A Review

**DOI:** 10.3390/mi13040630

**Published:** 2022-04-16

**Authors:** Chengkun Cai, Jian Wang

**Affiliations:** 1 Wuhan National Laboratory for Optoelectronics, School of Optical and Electronic Information, Huazhong University of Science and Technology, Wuhan 430074, China; chengkuncai@huat.edu.cn; 2 Optics Valley Laboratory, Wuhan 430074, China

**Keywords:** integrated optics, photonic chips, femtosecond laser fabrication, optical communications

## Abstract

Integrated optics, having the unique properties of small size, low loss, high integration, and high scalability, is attracting considerable attention and has found many applications in optical communications, fulfilling the requirements for the ever-growing information rate and complexity in modern optical communication systems. Femtosecond laser fabrication is an acknowledged technique for producing integrated photonic devices with unique features, such as three-dimensional fabrication geometry, rapid prototyping, and single-step fabrication. Thus, plenty of femtosecond laser-fabricated on-chip devices have been manufactured to realize various optical communication functions, such as laser generation, laser amplification, laser modulation, frequency conversion, multi-dimensional multiplexing, and photonic wire bonding. In this paper, we review some of the most relevant research progress in femtosecond laser-fabricated photonic chips for optical communications, which may break new ground in this area. First, the basic principle of femtosecond laser fabrication and different types of laser-inscribed waveguides are briefly introduced. The devices are organized into two categories: active devices and passive devices. In the former category, waveguide lasers, amplifiers, electric-optic modulators, and frequency converters are reviewed, while in the latter, polarization multiplexers, mode multiplexers, and fan-in/fan-out devices are discussed. Later, photonic wire bonding is also introduced. Finally, conclusions and prospects in this field are also discussed.

## 1. Introduction

In recent decades, integrated optics has developed rapidly due to its potential to meet the increasing capacity demands of communication networks. Since optical fibers largely replaced metallic wires in telecommunications, a number of optical integrated circuits also began to be produced for use in a variety of fields, such as photonics, microelectronics, laser technology and optical information processing [1]. In photonic chips, micro-optical devices such as lasers, optical modulators, optical (de)multiplexers, optical couplers, and optical detectors are integrated on the same substrate, which has great potential in modern optical communication applications [2,3,4]. Optical waveguides are the basic element of the photonic chip, which has two typical characteristics [5]. One is that the optical density in the waveguide is much greater than that of the bulk material, which enhances the laser characteristics and nonlinear characteristics of the material itself. Hence, better laser performance (low threshold, high gain and oblique efficiency) and more efficient nonlinear frequency conversion efficiency [6,7,8] can be achieved. The other is that the optical waveguide can guide the light wave to carry out long-distance diffraction-free propagation in the medium, forming and connecting optical elements to build a highly compact integrated optical path device [9].

Another technological advance that has encouraged the development of integrated optical devices in recent years is the availability of improved fabrication methods. Traditional optical waveguide fabrication methods, such as ion implantation [10], proton exchange [11], thin film deposition, metal ion diffusion [12] and so on, allow only fabrication of waveguide architectures on/near substrate surface, which can limit the transmission of light in only one direction. Although two-dimensional optical waveguides can also be fabricated by lithography mask technology, it requires optical designs with large dimensions/footprints and high complexity to ensure efficient optical connections, which is far from meeting the needs of the development of modern integrated optics. With the increasing integration and compactness of photonic devices, three-dimensional optical waveguides have become an inevitable development trend. Therefore, seeking a true three-dimensional optical waveguide fabrication method with high resolution, flexibility and processing quality is of great significance. With the continuous development of high-performance ultrafast laser technology, femtosecond laser fabrication technology has developed rapidly since the 1990s [13,14] and is widely used in basic and application research of many fields, especially providing a flexible and efficient true three-dimensional fabrication method for monolithic devices in integrated optics [15,16]. The ultrashort pulse width of femtosecond lasers greatly reduces the thermal expansion and heat dissipation effect in the processing process, and provides a necessary physical premise for obtaining high-precision spatial processing resolution [17]. With the help of three-dimensional or even six-dimensional electric moving platforms, femtosecond lasers can be used to design and fabricate three-dimensional photonics integrated devices with any configuration according to the actual needs [18]. In addition, the tightly focused femtosecond laser produces extremely high peak power at the focus, inducing a variety of nonlinear processes, such as multiphoton absorption, so that the laser can go deep into the interior of the material for three-dimensional processing beyond the diffraction limit [19]. The ubiquitous physical mechanisms make femtosecond laser fabrication a universal tool for a wide range of materials including glasses [20], crystals [21], polymers [22], and graphene [23,24,25,26]. Glasses, crystals, and polymers have been widely applied in this field for their diversity and universality. Graphene, as an advanced material, also attracts much attention in a wide range of areas due to its unique chemical and physical properties, such as high electrical conductivity, high mechanical strength, high thermal conductivity, low coefficient of thermal expansion and high optical transmittance [26]. Based on the optical waveguide structure fabricated by a femtosecond laser in transparent materials, a variety of passive or active waveguide devices in modern integrated optical systems can be realized. Femtosecond laser-fabricated active devices, such as waveguide lasers, waveguide amplifiers, electro-optic modulators, and nonlinear frequency converters, have been utilized in optical communication systems to realize laser generation, laser amplification, laser modulation, and frequency conversion [27,28]. Femtosecond laser-fabricated passive devices [29,30,31,32,33,34,35,36,37,38], especially polarization multiplexers, mode-multiplexers, and fan-in/fan-out devices can support multi-dimensional multiplexing in optical communication systems [39,40,41]. The photonic wire bonding technology based on polymers is one of the most promising practical advances of femtosecond laser fabrication, which solves issues regarding the connection of different photonic chips [42].

Several review articles on femtosecond laser-fabricated devices have been published [18,20,21,26,43,44]. Unlike these good reviews concentrated on devices based on specific materials, such as glasses, crystals, polymers, and graphene, this review is organized from the perspective of optical communications. Different components in optical communication systems have been realized by femtosecond laser fabrication in various materials. The fabrication of diverse waveguide configurations by femtosecond laser direct writing is introduced in Section 2. Section 3 focuses on the femtosecond laser-fabricated active devices including waveguide lasers, amplifiers, electric-optic (EO) modulators, and frequency converters. Section 4 focuses on the femtosecond laser-inscribed passive devices including polarization–division multiplexers, mode–division multiplexers, and fan-in/fan-out devices. In Section 5, photonic wire bonding for efficient optical coupling between different photonic integrated devices is introduced. An outlook with a summary will be given in Section 6.

## 2. Different Types of Waveguides Based on Femtosecond Laser Direct Writing

Femtosecond laser fabrication has been widely applied in the fabrication of waveguide devices in various materials. The femtosecond laser is usually focused by a microscopic objective onto the surface or into the bulk of samples to perform 3D precise fabrication. During the femtosecond laser direct-writing process, different physical mechanisms, such as multiphoton absorption, tunneling ionization, and avalanche ionization, are considered to be responsible for the generation of free electron plasmas, which could lead to localized modifications of materials [45,46,47].

The femtosecond laser-induced modifications can be divided into refractive index increasing modification and refractive index decreasing modification, which are jointly determined by the laser irradiation parameters and the properties of the transparent material itself. When the pulse energy exceeds the modification threshold slightly, a smooth refractive index change, referred to as Type I modification, is generated [48,49,50]. The sign of refractive index change can be either positive or negative according to the material itself. As the pulse energy rises to a high degree, the modification would turn up with severe damage in the materials, leading to a reduction in the refractive index, which is called Type II modification [51,52,53]. In the area around the tracks with Type II modification, an increment of refractive index occurs because of the stress field. Type II modification usually occurs in crystalline materials.

**Figure 1:**
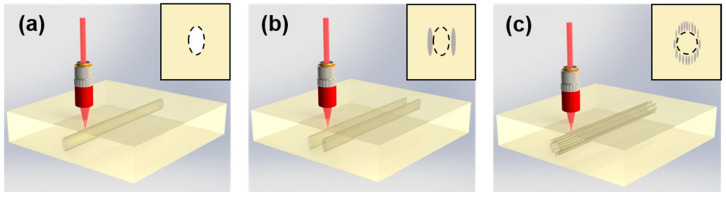
Schematic of the fabrication procedure of femtosecond laser-inscribed waveguides: (**a**) Single-line waveguide, (**b**) Double-line waveguide, and (**c**) Depressed cladding waveguide. The insets indicate the cross-sectional sketches of the waveguides. The shadows represent the fs-laser-induced tracks, and the dashed lines indicate the spatial locations of the waveguide cores.

Femtosecond lasers can manufacture optical waveguide structures with different morphological structures and dimensions in different materials. Based on the above two types of refractive index modification, the femtosecond laser-inscribed optical waveguides can be divided into the following three types. Based on the Type I modification, single-line waveguides can be formed in irradiated regions with the increased refractive index [54], as shown in Figure 1a. This positive change mechanism is very common in amorphous materials, e.g., in a majority of glasses [55,56,57]. This kind of optical waveguide with a simple structure and an efficient and convenient fabricating process easily forms three-dimensional optical waveguide devices. Combined with laser multiple scanning technology, the symmetrical waveguide structure with an arbitrary size can be realized by arranging a certain number of laser writing scratches in space. In crystals, however, single-line waveguides have been realized in only a handful of hosts, e.g., LiNbO_3_ [58]. The physical properties of the crystal material, such as laser characteristics and nonlinear characteristics, would be damaged due to the femtosecond laser direct radiation. Moreover, this kind of optical waveguide is polarization-dependent, which only supports the transmission of light waves in a specific polarization direction. Based on the type II modification, the area between the writing lines possesses a relative higher refractive index due to the stress-field, which is called double-line waveguide [52,53], as shown in Figure 1b. Without laser irradiation in the light guiding region, the laser and nonlinear characteristics of the crystal itself are well preserved. The performance remains stable at high temperatures. The structure is suitable for most crystal materials. However, the effective distance between the two laser-induced traces of this kind of optical waveguide is limited (10–30 μm), which is not conducive to the coupling of larger cross-sections. Such waveguides still suffer from the same polarization-dependent guiding as the single-line ones, which makes the realization of some nonlinear optical devices challenging. Additionally, a depressed cladding waveguide [59,60,61,62], which is surrounded by a number of tracks with Type II modification, can support guidance along both TE and TM polarizations, as shown in Figure 1c. In principle, arbitrary geometries of optical waveguide structure can be fabricated by arranging and combining multiple parallel lines flexibly. In practice, a circular shape with adjustable diameter size is preferred because it fits well to the optical fibers, and it can support fundamental or high-order modes in an individual waveguide. Since the guiding core in such structures is beyond the area of the laser-induced damages, most of the advantages that double-line waveguides possess also apply to cladding waveguides. However, since its relative complexity and large size in fabrication, depressed cladding waveguide fabrications are usually quite challenging and time-consuming.

## 3. Active Devices

**Figure 2:**
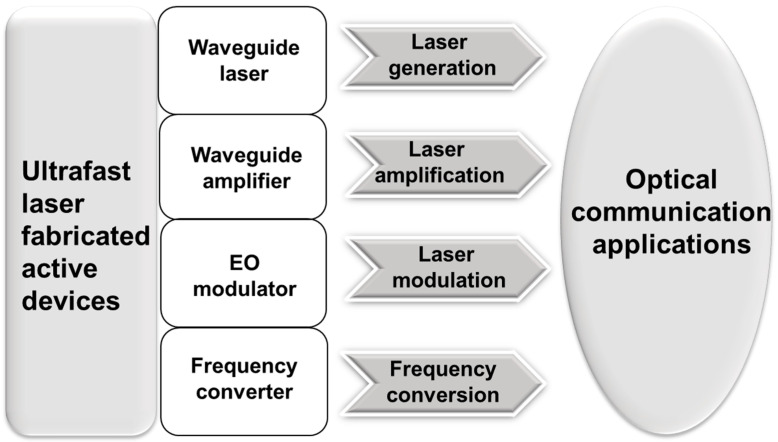
Femtosecond laser-fabricated active devices for optical communication applications.

Femtosecond laser fabrication has been applied to several active materials, and some of them resulted in being suitable to demonstrate active photonic devices. As shown in Figure 2, active devices, such as waveguide lasers, waveguide amplifiers, EO modulators and frequency converters, have been fabricated by femtosecond laser direct writing. Additionally, these active devices have been used in optical communication applications with diverse functions including laser generation, laser amplification, laser modulation and frequency conversion.

### 3.1. Waveguide Lasers

The femtosecond laser fabrication technique has already been successfully utilized to create waveguide lasers based on various rare-earth doped glasses and crystals. The rare-earth doped structures provide advantageous spectroscopic properties of the waveguide lasers. High optical efficiency with reduced heat generation can also be obtained due to the low quantum defects of these materials, which is extremely useful for the realization of compact, high-power devices. Furthermore, the waveguide configuration provides excellent beam confinement and good spatial overlap between the pump and signal beams, resulting in higher lasing efficiencies and lower pumping thresholds [63].

#### 3.1.1. Waveguide Lasers Based on Glass

Femtosecond laser-inscribed waveguide lasers have been reported in a range of different rare-earth doped glass hosts, such as Er:Yb-doped phosphate glass [64], Yb-doped bismuthate glass [63], Er:Yb-doped oxyfluoride silicate glass [49], Ho-doped fluorozirconate glass [65] and Yb-doped silicate glass [66]. A broad range of laser spectrum ranging from 1 µm to the mid-infrared has been achieved.

Ytterbium-doped medium, which emits laser wavelengths of around 1 micron, is anideal medium for high-power direct diode pumping with a low thermal load. Yb:ZBLAN (ZrF4-BaF2-LaF3-AlF3-NaF) and $\mathrm{Yb}:{\mathrm{IOG}}_{10}$ waveguide lasers fabricated by the femtosecond laser direct writing technique were reported [66]. Multiple negative refractive index modifications were aligned in a depressed cladding geometry to create waveguides in both glasses. In Yb:ZBLAN glass, the high laser slope efficiency of 84% with a maximum output power of 170 mW at 1030 nm was demonstrated. By using $\mathrm{Yb}:{\mathrm{IOG}}_{10}$, a laser performance of 25% slope efficiency and 72 mW output power at 1030 nm was achieved.

There is also study pointing out the outstanding properties of Yb-doped bismuthate glasses, such as their large fluorescence lifetimes, large absorption and emission cross sections, low quantum defects, moderate cooperative absorption probabilities, and broad fluorescence bands [63]. Laser slope efficiencies close to the quantum defect limit and in excess of 78% have been obtained from a femtosecond laser-inscribed waveguide laser in the ytterbium-doped bismuthate glass.

**Figure 3:**
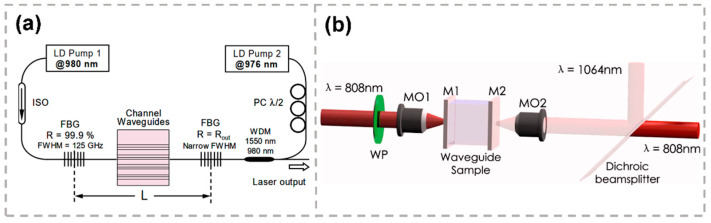
(**a**) The setup of waveguide linear laser cavity with a bi-directional pumping scheme adopted [48]. (**b**) Schematic of the experimental setup for the waveguide laser generation. WP: wave plate; MO1 and MO2: microscope objective lens; M1 and M2: laser cavity mirrors adhered to the two end-facets of the sample [68]. Reproduced from [48] with permission of the Optica Publishing Group. Reproduced from [68] with permission of the AIP Publishing, 2022.

Lasers at 1.5 μm are essential tools for optical communications applications. A 20 mm-long waveguide laser was fabricated on an Er:Yb-doped phosphate glass by a femtosecond laser. The output power of 1.7 mW with 300 mW of pump power was obtained at 1533.5 nm [64]. A more compact and efficient single longitudinal mode laser at 1.5 μm based on a femtosecond laser-inscribed erbium–ytterbium-doped phosphate waveguide was also demonstrated [48], as shown in Figure 3a. The maximum output power exceeding 50 mW with 21% slope efficiency was measured in single longitudinal and transverse mode operation. The active medium was a 20 mm-long single-line waveguide fabricated in a phosphate glass substrate doped with 2 wt.% of Er2O3 and 4 wt.% of Yb2O3. Dopant concentrations and sample length have been optimized to obtain high gain per unit length. The waveguide laser cavity employed a linear configuration, and the active waveguide was butt-coupled on both sides to fiber Bragg gratings (FBGs). On one side, a broad-band flat top FBG with 1 nm bandwidth (FWHM) was used to provide high reflectivity (99.8%). On the other side, a narrow-bandwidth FBG with about 0.1 nm FWHM was used as output coupler. An index-matching fluid is inserted between waveguide and fiber ends to support high power density at 980 nm.

Fluor-zirconate glasses, in particular ZBLAN, have attracted a great deal of attention for laser applications beyond 1 μm wavelength due to their material properties of outstanding WG circularity and excellent reproducibility of the laser written modifications. A femtosecond laser-inscribed depressed cladding waveguide laser on ${\mathrm{Tm}}^{3+}$-doped ZBLAN glass that produces 47 mW at 1880 nm with a 50% internal slope efficiency was reported [67]. Furthermore, a large mode-area holmium-doped ZBLAN waveguide laser operating at 2.9 μm was also reported [65]. The laser was also based on ultrafast laser-inscribed depressed cladding waveguides fabricated in uniformly rare-earth-doped bulk glass. It had a threshold of 28 mW and produced up to 27 mW of output power at an internal slope efficiency of approximately 20%.

#### 3.1.2. Waveguide Lasers Based on Crystals

Waveguide lasers have also been realized in a couple of crystalline gain materials, e.g., Nd or Yb-doped YAG [52,62,69,70,71,72], Nd-doped ${\mathrm{YVO}}_{4}$ [68], Nd-doped KGW [73], and Ti:Sapphire [74].

A continuous-wave 1064 nm laser generation from a femtosecond laser-inscribed waveguide in $\mathrm{Nd}:{\mathrm{YVO}}_{4}$ with pumping at 808 nm was reported. Single-mode stable laser operations had been observed with pump powers at thresholds as low as 14 mW [68]. The waveguide laser experiments were performed by using an end-face coupling system as shown in Figure 3b. A 20× microscope objective lens was used to focus the pump light beam into the waveguides. The generated laser beam at 1064 nm was collected by another 20× microscope objective from the output facet of the waveguide and separated from the residual pump through a dichroic beam splitter. When the laser gain experiments were performed with cavity mirrors (a mirror with transmission of 98% at 808 nm and reflectivity 99% at 1064 nm for the front face, and a mirror with reflectivity 99% at 808 nm and 95% at 1064 nm as the output coupler), the output coupler efficiency of the composed laser cavity was 5%. In the case without using any laser mirrors, the laser generation can still be realized by directly using the two polished facets with Fresnel reflection. The transmittance of the waveguide facets can be estimated to be close to 90%. Laser slope efficiencies as high as 38.7% with a maximum output light power of about 9.5 mW was obtained. Mirrorless waveguide laser is one of the most straightforward solutions toward monolithic on-chip photonic integration in robust and compact packages [68].

Nd:YAG crystal is also widely used for solid-state lasers due to its outstanding fluorescence and thermal and mechanical properties. A rectangular Y-branch depressed cladding waveguide laser has been fabricated in Nd:YAG crystals by femtosecond laser direct writing. Continuous wave laser generation at a wavelength of 1.06 μm has been achieved under the optical pump at 808 nm. When the splitting angle of Y-branch waveguides reached 0.5°, the maximum output power of 0.2 W with a slope efficiency of 20% was obtained [72]. The Y-branch splitter can serve as the basic element to realize the wavelength division multiplexing in the optical networks.

**Figure 4:**
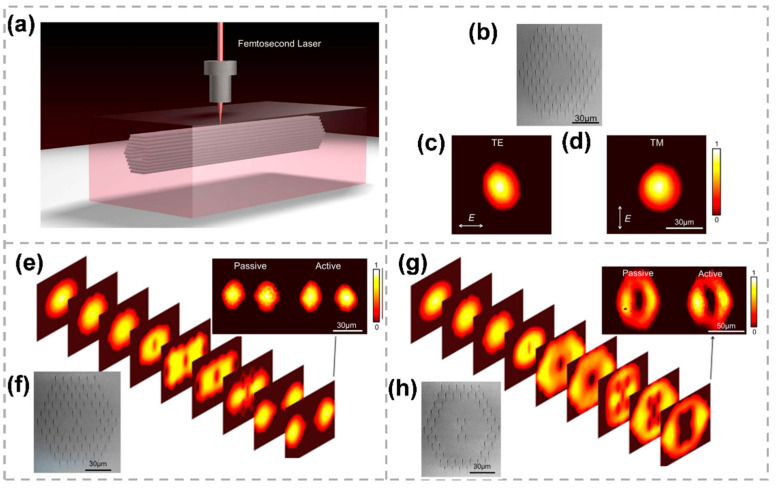
(**a**) The fabrication schematic plots of a typical photonic microstructure with a guiding core surrounded by a hexagonal track array. (**b**) Microscopic image of the cross section of the femtosecond laser written photonic fundamental mode waveguide in Nd:YAG crystal. Measured near-field modal profiles along both TE (**c**) and TM (**d**) polarizations at 1064 nm. (**e**) Beam profile evolution as the 1064 nm light propagates along the splitter waveguide structure. (**f**) Microscopic pictures of splitter waveguide structure. (**g**) beam profile evolution as the 1064 nm light propagates along the ring-shaped photonic structure. (**h**) Microscopic pictures of the ring-shaped photonic structure [70]. Reproduced from [70] with permission of the Springer Nature, 2022.

Benefiting from the flexible geometries, sizes and refractive index profiles defined by femtosecond laser direct writing, the depressed-cladding waveguides are capable of supporting excellent single- as well as multi-mode light. Furthermore, a novel family of three-dimensional (3D) photonic microstructure lasers monolithically integrated in a Nd:YAG crystal wafer by femtosecond laser direct writing was demonstrated. They are capable of simultaneous light generation, waveguiding, and manipulation. The waveguides with multiple configurations and geometries are fabricated by a femtosecond laser with the unique feature of flexible 3D micro-engineering, as shown in Figure 4a. The microscopic image of the cross section of the femtosecond laser-written photonic fundamental mode waveguide is shown in Figure 4b. Figure 4c,d display the measured near-field modal profiles along both TE (Figure 4c) and TM (Figure 4d) polarizations at 1064 nm. By changing the cross-sectional topography of the waveguide, the light-beam manipulation of beam splitting and ring-shaped transformation are achieved, as shown in Figure 4e–h. The integration of thin-layer graphene as a saturable absorber in the 3D laser structures allows for efficient passive Q-switching of tailored laser radiations, which may enable miniature waveguiding lasers for broader applications. By using the direct pump of the monolithic integrated structures, waveguide lasing has been obtained in both continuous wave (CW) and pulsed (passively Q-switched by graphene) regimes [70].

### 3.2. Waveguide Amplifiers

To increase the number of channels in the wavelength-division multiplexing (WDM) system, amplifiers with broad and flat gain regions are crucial components for optical communication. Femtosecond laser-inscribed waveguide amplifiers have been fabricated in various RE-doped glass systems: Yb-doped phosphate glass (Kigre QX), Er-Yb co-doped phosphate glass (Kigre QX), Nd-doped silicate glass, Er-doped oxyfluoride silicate glass, and Er-Yb co-doped oxyfluoride silicate glass [75,76,77,78]. The first active waveguide fabricated by a femtosecond laser was in Nd-doped glass, which is very efficient four-level active media, but unsuitable for application at telecom wavelengths. Therefore, other active glasses, especially Er-doped and Er-Yb co-doped glasses allowing operation in the C band, have attracted much attention.

Phosphate glasses have the advantages of a broadband emission spectrum around 1.5 μm, long lifetime of the Er metastable state, and high phonon energies allowing very efficient pumping. Hence, an optical waveguide on erbium–ytterbium-doped phosphate glass with an appreciable net gain in the full C-band of optical communications was fabricated by femtosecond laser direct writing [75]. The 37 mm-long amplifier was butt-coupled on both sides to single-mode fibers (SMFs), using an index-matching fluid to reduce coupling loss. The peak internal gain of 9.2 dB was obtained at 1535 nm, and a minimum internal gain of 5.2 dB was obtained at 1565 nm. The relatively low insertion losses of 1.9 dB in the system enable an appreciable net gain in the full C-band of optical communications for the first time.

Erbium-doped oxyfluoride silicate glass combines the promising spectroscopic properties of fluoride glasses (similar to phosphate) with the structural stability and compatibility of silicate glasses [76]. A waveguide amplifier in this oxyfluoride silicate glass was fabricated with a total background insertion loss excluding absorption of only 1.2 dB. A maximum net gain of 1.4 dB in the C band (1529–1561 nm) was observed in the 10 mm-long waveguide [77]. Other materials, such as bismuthate (${\mathrm{Bi}}_{2}O_{3}$) glass, may also be suitable for Er-doped waveguide amplifiers while providing additional benefits of a wide and flat gain spectrum and the ability to be doped with sufficient concentrations of Er3+ ions. An Er-doped bismuthate glass waveguide amplifier fabricated by femtosecond laser direct writing has been reported. When pumped with 1050 mW of 980 nm light, the amplifier exhibited a peak internal gain per unit length of 2.3 dB/cm at 1533 nm and a peak fiber-to-fiber net gain of 16.0 dB at 1533 nm.

**Figure 5:**
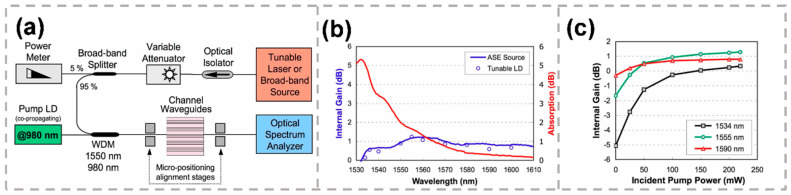
(**a**) Experimental setup for waveguide amplifier characterization. (**b**) Measured absorption spectrum (red) of the 25 mm-long active waveguide and internal gain (blue solid line and circles) at 200 mW incident pump power. (**c**) Internal gain at 1555 nm (circles), 1534 nm (squares) and 1590 nm (triangles) as a function of incident pump power [78]. Reproduced from [78] with permission of the Optica Publishing Group, 2022.

To broaden the emission bandwidth compared to phosphate glasses, tellurite-modified phosphates have been recently proposed to extend the amplification into the L band (1565–1625 nm). Waveguide amplifiers on Er-doped tellurite glass modified by phosphate have also been fabricated by femtosecond direct writing [78], as shown in Figure 5a. Figure 5b displays the measured absorption spectrum of the 25 mm-long active waveguide and internal gain at 200 mW incident pump power. A maximum internal gain of 1.25 dB at 1555 nm was demonstrated, as well as internal gain in the whole C+L band. The small-signal internal gain as a function of incident pump power at 1534 nm, 1555 nm and 1590 nm signal wavelengths is shown in Figure 5c. Phosphate modification induced a positive index change in tellurite glass by femtosecond laser writing. The optical waveguide amplifier in phospho-tellurite glass provided a large internal gain bandwidth of 100 nm, spanning the entire C and L bands (1530–1630 nm) [56].

It is worth pointing out that another promising active substrate, the Bi-doped silica glass [79], can exhibit broadband emission around 1.3 μm. A high-quality waveguide amplifier of 1.7 cm inside Bi-doped silicate glass was fabricated by femtosecond laser direct writing. A broadband fluorescence with a bandwidth (FWHM) of up to 500 nm centered on 1.3 μm was obtained.

### 3.3. EO Modulator

EO modulators are designed to modulate the phase, amplitude, intensity and polarization of light with the external voltage. Femtosecond laser-fabricated EO modulators based on a Mach–Zehnder interferometer (MZI) [80,81,82,83,84], waveguide Bragg grating [85,86] and a microresonator [87,88] have been reported. Additionally, lithium niobate (LiNbO_3_) is widely used in optoelectronics due to its large transmission window and excellent nonlinear properties [86].

**Figure 6:**
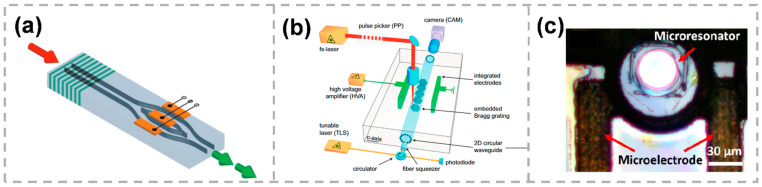
(**a**) Schematic of a hybrid fs laser-written chip including an amplitude modulator [83]. (**b**) Schematic of the experimental setup for direct integration and characterization of waveguide embedded Bragg gratings (WBG) in LiNbO_3_ [86]. (**c**) The top view of the EO tunable LiNbO_3_ microresonator integrated with in-plane microelectrodes [88]. Reproduced from [83] with permission of the John Wiley and Sons, 2022. Reproduced from [86] with permission of the Optica Publishing Group, 2022. Reproduced from [88] with permission of the Springer Nature, 2022.

MZI-based EO modulators are composed of a MZI and metal electrodes either coated on the substrate surfaces or embedded in femtosecond laser ablated grooves [83], as shown in Figure 6a. The effective EO coefficient $r_{33}$ in a Type-I-based single-line LiNbO_3_ (x-cut) waveguide has been found to be only half of the original value, leading a degraded performance of EO behavior. Thus, type-II-based double-line geometries in MZI-based EO modulators are more common. The extinction ratios (ERs) of 9 dB with a half-wave voltage Vπ of 19 V at a wavelength of 632.8 nm, 11 dB (Vπ of 23 V) at 532 nm, and 11 dB (Vπ of 45 V) at 1.55 μm have been reported. The performances are unsatisfied due to the non-optimized EO overlap and the high bending losses. For further improvement, waveguide of low loss and the optimization of EO overlap are urgently needed.

Periodic refractive index changes embedded into waveguides, which can exhibit spectral frequency selectivity, have been applied to EO modulators. The EO first-order waveguide Bragg grating in a double-line LiNbO_3_ waveguide has been reported [85]. The distance between waveguide lines was adjusted from 7 μm to 15 μm to support sufficient guiding at 1550 nm. The first-order grating was fabricated by separating refractive index voxels with a periodicity of 349.6 nm. The waveguide was covered by copper electrodes from the top and bottom faces and connected to a high-voltage power supply unit to achieve an electro-optic response. A maximum shift in the reflection peak of 625 pm was observed with the external electrical field increasing from −22 to +22 V/μm.

Furthermore, the EO modulator waveguide Bragg grating was improved by taking a hybrid design of a circular type-II waveguide and a multi-scan type-I Bragg grating [86]. The type-II waveguide geometry was fabricated to provide low loss, symmetric guiding with a superior mode fidelity, and the largest electro–optic coefficient r33 for extraordinary polarization. Inside the type-II waveguide, type-I Bragg gratings were inscribed with a period of Λ = 704 nm by using the multi-scan technique, as shown in Figure 6b. The measured maximum peak shift was 1180 pm, with the applied voltage ranging from −840 to +840 V [85].

High-Q on-chip LiNbO_3_ microresonators based on the femtosecond laser direct writing technique have been reported [88]. The metallic microelectrodes were first fabricated on the substrate by using femtosecond laser0assisted selective electroless copper plating, as shown in Figure 6c. Then, the microresonator located between the microelectrodes was further fabricated by femtosecond laser direct writing accompanied by focused ion beam milling. The experimental results indicate that the tuning coefficient of 3.41 pm/V can be reached with the external voltage above 80 V.

A higher effective mechanical quality ($Q_{m}$) factor of 2.86 × 10^8^ in a lithium niobate microresonator fabricated by femtosecond laser direct writing followed by chemo-mechanical polishing have been reported [87]. Additionally, the real-time electrical tuning of the optomechanical frequency with an electro-mechanical tuning efficiency around −134 kHz/100 V was measured, suggesting a great potential for a broad range of applications in metrology, sensing, information processing and quantum physics.

### 3.4. Frequency Converters

Waveguide-based frequency converters exhibit superior performance compared to their bulk counterparts due to the non-diffraction beam over a long distance in the waveguide and high optical intensity. Frequency conversion processes, mainly second harmonic generation (SHG), have been demonstrated in various femtosecond laser-inscribed waveguide frequency converters. Meeting the phase-matching condition is a precondition to achieve efficient SHG, by either birefringence phase matching (BPM) [89] or quasi phase matching (QPM) [90,91,92,93,94,95].

The SHG can be generated using BPM in standard crystals, which are cut along a specific orientation. Lithium niobate (LiNbO_3_) is widely used in nonlinear optics because of its large electro-optic and nonlinear coefficients and availability in high optical quality [89,96,97]. Frequency doubling of 1064 nm radiation was reported utilizing birefringent phase matching in a Lithium niobate waveguide fabricated by femtosecond laser direct writing [89]. The LiNbO_3_ sample doped with 7 mol% MgO was used to avoid the degeneration of waveguide properties caused by photorefractive effects with the generated green laser light. To achieve phase matching, the propagation constants of the fundamental and second harmonic modes were designed to be equal. For BPM, the double-line waveguide was fabricated along the y direction in x-cut LiNbO_3_ to ensure a propagation perpendicular to the optic axis. A suitable temperature which depends on the crystal stoichiometry and doping was also chosen to achieve maximal SHG efficiency. The maximal conversion efficiency of 49% was obtained with an input peak power of 480 W. The normalized conversion efficiency was 0.6%/(${W\;\mathrm{cm}}^{2}$).

**Figure 7:**
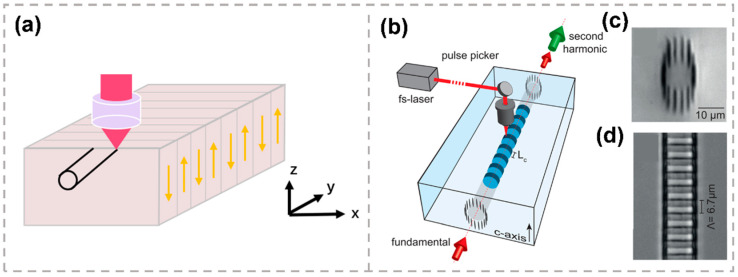
(**a**) Schematic of the femtosecond laser direct writing setup for fabrication of the waveguide in PPLN [90]. (**b**) Schematic of the QPM waveguide design in z-cut lithium niobate [94]. (**c**) Optical microscope images the circular waveguide structure [94]. (**d**) top view of the multi-scan grating section with a period of 6.7 μm [94]. Reproduced from [90,94] with permission of the AIP Publishing, 2022.

Compared to the relatively low nonlinear coefficient and the instability in time and temperature for BPM, QPM in periodically domain-inverted crystals, such as periodically poled LN (PPLN), exhibits huge advantages of the large coefficient d33 and the ability to arbitrarily choose the phase matching wavelength and temperature. Frequency doubling with QPM has been reported in PPLN single-line waveguides [90]. However, single-line waveguides in PPLN yield a reduced nonlinearity. Additionally, single-line waveguides are only suitable for low input powers because they can be thermally annealed at 150 °C. To achieve more efficient SHG, the fabrication of thermally stable double-line waveguides in Z-cut PPLN crystals utilizing femtosecond lasers has been demonstrated. The 10 mm-long Z-cut PPLN crystal sample was fabricated using the external pulse field poling technique with a QPM period of 18.6 um, as shown in Figure 7a. The normalized efficiency of 4.8%/(${W\;\mathrm{cm}}^{2}$) was obtained at a QPM wavelength of 1548.2 nm and at an optimal temperature of 150.4 °C.

The QPM structure can also be fabricated by femtosecond laser direct writing. Instead of domain inversion, the nonlinearity is damped after laser modification, which reduces the phase contributions of the SHG process. The efficiency of such a laser-induced quasi phase-matching waveguide is below that of PPLN fabricated by the external pulse field poling technique. However, the laser-induced quasi phase-matching waveguide can be fabricated in a single monolithic process, which provides immediate compatibility to integrated optical elements. SHGs of 1064 nm radiation in quasi phase-matched waveguide structures fabricated by femtosecond laser direct writing have been demonstrated [94]. The standard femtosecond laser direct writing setup to fabricate the waveguides is illustrated in Figure 7b. A cladding waveguide with circular cross section based on type-II modifications is shown in Figure 7c. The QPM waveguide with a phase-matching period of 6.7 μm is shown in Figure 7d. The grating is inscribed by the multi-scan technique with high transverse resolution of 700 nm in both directions. A maximum conversion efficiency of 5.72% was obtained for a 6 mm-long femtosecond laser-inscribed QPM waveguide.

## 4. Passive Devices

**Figure 8:**
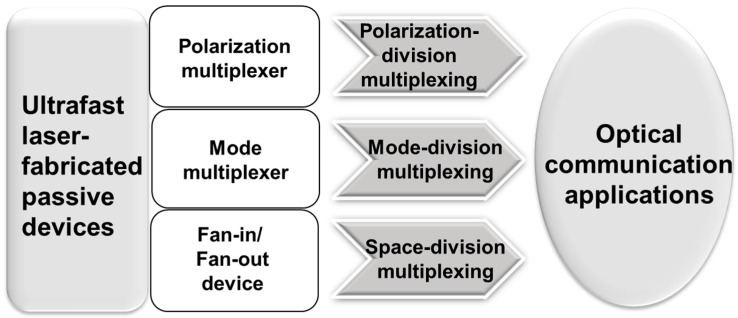
Femtosecond laser-fabricated passive devices for optical communication applications.

With the popularization of cloud-computing-related applications and the development of virtual reality and artificial intelligence, the demand for network capacity is explosively increasing. To meet this demand, optical fiber communication systems are evolving from multi-channel and high-speed to ultra-high-speed, ultra-large capacity and ultra-long distance. On-chip multiplexing technology is a common method to solve the problem of high transmission rate, which can reduce the cost and facilitate later maintenance by integrating multiple channels into one optical fiber for transmission. At present, there are three multiplexing technologies in the physical layer: space-division multiplexing (SDM) [98,99,100], polarization-division multiplexing (PDM) [101] and wavelength-division multiplexing (WDM) [102,103]. Space-division multiplexing technology includes multi-core division multiplexing (MCF) [41] and mode-division multiplexing (MDM) [104,105,106,107,108]. As shown in Figure 8, passive devices, such as polarization multiplexers, mode multiplexers, and fan-in/fan-out devices have been fabricated by femtosecond laser direct writing in applying PDM, MDM and SDM in optical communications.

### 4.1. Polarization Multiplexers

The birefringence effect of the waveguide produces transverse electric (TE) field and transverse magnetic (TM) field, which are orthogonal and independent from each other. Polarization division multiplexing technology, with two orthogonal polarization states used for signal multiplexing/demultiplexing, can double the system capacity. Depending on the material and the irradiation conditions, birefringence in femtosecond laser modification (typical values rang in 10^−6^–10^−4^) can originate from one or more of the following sources, such as the laser-induced periodic nano-structures, the asymmetric mechanical stresses induced in the focal region, or the ellipticity of the written waveguide cross section [39,109,110,111,112,113,114,115,116]. Hence, through different fabrication parameters, femtosecond lasers can flexibly change the birefringence effect of waveguides, so as to manufacture various polarization-sensitive or polarization-insensitive devices [111].

**Figure 9:**
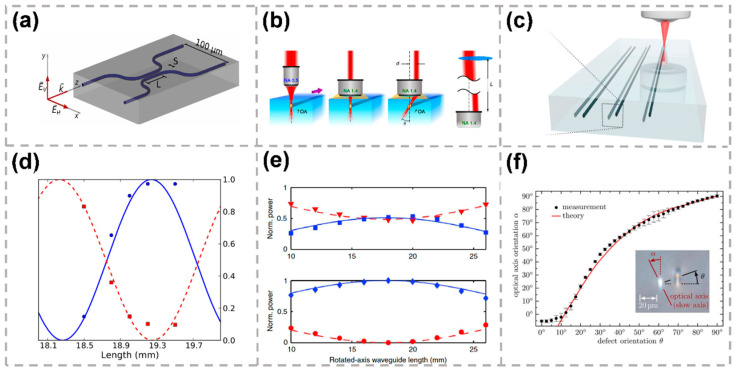
(**a**) Schematic of the integrated polarization directional coupler (PDC) [110]. (**b**) Femtosecond laser writing of tilted integrated waveplates [115]. (**c**) Sketch of the writing setting, with which the waveplates are fabricated [109]. (**d**) Measured coupling ratio, r, as a function of coupling length for vertical polarized (blue circle) and horizontal polarized (red square) modes together with calculated fits (solid and dashed lines) [110]. (**e**) For horizontally polarized input light, the measured normalized power transferred into the horizontal/vertical polarization states and diagonal/antidiagonal polarization states is reported [115]. (**f**) Experimental data and best fit model of the reorientation of the optical axis as a function of the azimuthal position of the defect [109]. Reproduced from [110] with permission of the Optica Publishing Group, 2022. Reproduced from [109,115] with permission of the Springer Nature, 2022.

Laser-induced nanogratings contribute a strong-form birefringence effect for waveguides fabricated in fused silica [110]. The orientation of nanogratings is linear and oriented to be parallel or perpendicular with respect to the scanning direction by using a half-wave plate. For parallel and perpendicular writing polarizations, the form birefringence values were measured to be (5.2 ± 0.5) × 10^−5^ and (2.1 ± 0.1) × 10^−4^ at a 1550 nm wavelength, respectively. By finely tunning the laser exposure parameters, waveguides with special wave retardance and polarization-dependent coupling with minimal optical losses can be examined and optimized in the 1250 nm to 1700 nm spectrum domain. Furthermore, zero-order quarter-wave and half-wave retarders together with polarization beam splitters were demonstrated. As shown in Figure 9a, polarization-splitting directional couplers were designed and demonstrated with −19 dB and −24 dB extinction ratios for the polarization splitting [39]. The measured coupling ratio of directional couplers fabricated with S = 8 μm waveguide separation is shown in Figure 9d as a function of interaction length.

Generally speaking, the polarization birefringence direction is fixed along the transmission direction of femtosecond laser. However, by tilting the transmission direction of the writing beam, the inscribed waveguide optical axis can be rotated as well, thus changing the polarization birefringence direction [115], as shown in Figure 9b. In the experiment, the laser beam impinges on the objective aperture in an off-center way, leading the beam propagates in the substrate at an angle with the focus position not altered. For horizontally polarized input light, the measured normalized power transferred into the horizontal/vertical polarization states and diagonal/antidiagonal polarization states is displayed in Figure 9e. The fabricated waveguide will then have a tilted cross-section, resulting in a rotated optical axis. Hence, femtosecond laser-inscribed waveguide-based optical waveplates, with arbitrarily rotated birefringence axis, are also presented.

Moreover, the intrinsic birefringence of the femtosecond laser-inscribed waveguides is usually weak. Therefore, the polarization beam splitter (PBS) composed of such waveguides needs a larger size to realize the corresponding functions. A method to decrease the size of the PBS is proposed. By shortening the distance between the coupled waveguides, strong anisotropic mechanical stress was introduced to increase the polarization birefringence of the two waveguides. When the distance between the waveguides is below 5 μm, a PBS with an order of magnitude lower interaction length of 3.7 mm is demonstrated. The extinction ratios for the horizontal and vertical polarizations are 16 dB and 20 dB, respectively [112].

In addition, the optical axis of the birefringent waveguide can also be rotated due to the artificial stress. The stress can be introduced to the waveguide by writing an additional modification track near to the waveguide, as shown in Figure 9c [109]. By adjusting the length of the track along the waveguide, the retardation between ordinary and extraordinary field components can be precisely tuned to realize half-wave plate and quarter-wave plate operations. The orientation of the optical axis is a function of the relative position of the two guides. Thus, arbitrary desired wave-plate operations in the waveguide can be achieved. The orientation of the optical axis as a monotonous function of the azimuthal angle h of the track is plotted in Figure 9f. When the azimuthal angle of the track increases, the tilt of the waveguide’s optical axis also increases and reaches a maximum of $\propto_{max}\;$= 90° for θ = 90°. The main advantage of this approach is that the original waveguide shape remains unchanged when the optical axis’ orientation is changed, leading to an additional loss in the presence of the track less than 0.01 dB/cm.

Different from the above method with defect tracks written nearby the waveguide, another method with a pair of tracks laid out quite close with a bit overlapped was proposed [113]. The track pairs can still guide light behaving as a single-mode waveguide with an optimal separation without energy dissipation into the defect track. A precise change in relative position in the track pairs can induce the artificial asymmetric distribution of the refractive index, generating a rotation of the birefringent optical axis. A rotated polarization directional coupler with the two axis-rotated waveguides was obtained with the extinction ratios on average about 16 dB and 20 dB for the corresponding orthogonal polarizations, respectively.

### 4.2. Mode Multiplexers

In order to overcome the current limitation of the transmission capacity in the single-mode fiber (SMF) systems, mode-division multiplexing (MDM) technology, one of the promising approaches in space-division multiplexing technology, has been intensively investigated by multiplexing independent signals into the spatial modes acting as separate paths in a multi-mode fiber (MMF). Several kinds of mode multiplexers have been proposed to achieve MDM transmission. Free-space optics using phase plates or spatial light modulators can excite the spatial modes directly. However, it requires large bulk optics, and the insertion loss may increase with the number of modes. Mode multiplexers based on optical fiber or waveguide can be a more practical approach due to intrinsic low loss and compactness. Among of them, mode multiplexers based on femtosecond laser-inscribed 3D waveguides attract more and more attention due to the features of 3D processing ability and flexibility. It includes mode multiplexing by cascaded mode selective couplers applying the coupling between the waveguide modes and photonic lanterns, bringing the waveguide core closer together. Mode multiplexers based on mode-selective couplers (MSC) and mode multiplexers based on photonic lantern are discussed here.

#### 4.2.1. Mode Multiplexers Based on Mode-Selective Couplers

**Figure 10:**
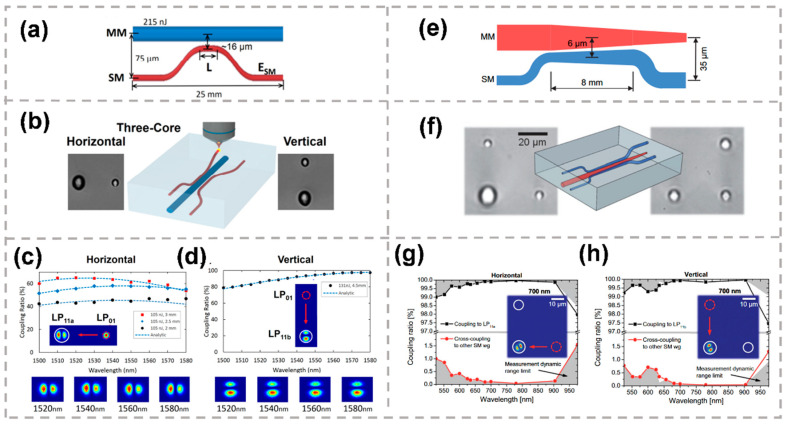
(**a**) Schematic of the (**a**) horizontal and vertical two-couplers, each comprising a multimode and single-mode core [118]. (**b**) Brightfield microscope images of the end-faces (writing laser incident from the top) and 3D sketch of the fabricated coupler [119]. The measured coupling ratios for the (**c**) horizontal and (**d**) vertical couplers as compared with approximate analytic models. The coupling ratios of two other horizontal couplers written using the same pulse energy but with different interaction length are also shown. The $LP_{11}^{a}$ and $LP_{11}^{b}$ mode profiles are shown in the inset [119]. (**e**) Schematic of a two-core tapered mode-selective coupler comprising a tapered multimode and a counter-tapered single-mode waveguide [119]. (**f**) Brightfield microscope images of the end-faces (writing laser incident from the top) and 3D sketch of the fabricated coupler [119]. (**g**) Coupling and cross-coupling ratios when injecting light into the horizontal single-mode waveguide as a function of wavelength [119]. (**h**) Coupling and cross-coupling for light injection into the vertical single-mode waveguide [119]. Reproduced from [118] with permission of the Optica Publishing Group, 2022. Reproduced from [119] with permission of the John Wiley and Sons, 2022.

An MSC is a directional coupler that provides selective mode coupling between the fundamental mode of a single-mode waveguide and a higher-order mode of a few-mode waveguide [117]. Figure 10a displays the schematic of the horizontal and vertical two-couplers, each comprising a multimode and single-mode core. A femtosecond laser-inscribed mode multiplexer composed of consecutive mode selective couplers was proposed [118]. The structure of the fabricated coupler is shown in Figure 10b. As shown in Figure 10c,d, horizontally and vertically written two-core couplers are designed to allow for the multiplexing of the $LP_{11}^{a}$ and $LP_{11}^{b}$ spatial modes of an optical fiber, respectively. The excellent mode extinction ratios (25–37 + dB) and low loss (~1 dB) between 1500 and 1580 nm were obtained. The mode-selective functionality was achieved by matching the modal propagation constants. By adjusting the average power of the laser, the waveguide size can be adjusted flexibly. The core diameters were chosen to make sure that the propagation constants of the fundamental $LP_{01}$ modes in the horizontal and vertical single-mode cores matched those of the respective orthogonal $LP_{11}^{a}$ and $LP_{11}^{b}$ modes in the multimode core. When written in sequence, the couplers allow for the multiplexing of all $LP_{01}$, $LP_{11}^{a}$ and $LP_{11}^{b}$ modes, forming a single three-dimensional three-core mode multiplexer.

The mode-selective couplers above rely on fastidious phase-matching and interference to achieve their functionality. To simplify the phase-matching condition, tapered couplers are presented, which can achieve high mode conversion efficiency with phase matching at a certain position in the conical coupling region. Figure 10e displays the schematic of a two-core tapered mode-selective coupler comprising a tapered multimode and a counter-tapered single-mode waveguide. A mode multiplexer based on fully pig-tailed tapered velocity couplers fabricated by femtosecond laser direct writing was demonstrated [119]. The structure of the fabricated coupler is shown in Figure 10f. Tapering of the waveguides was accomplished by linearly changing the pulse energy of the femtosecond laser with a computer-controlled attenuator. Unlike the standard mode coupler, it is not necessary to meet the precise phase-matching conditions on the extended length, nor to reduce the coupling coefficient to zero under the specified coupling length because any significant interaction has long ceased near the end of the device. The precise region of the taper over which the coupling takes place is also immaterial. For this reason, small shifts in the propagation constant crossover point along the device caused by changes in wavelength or the dimensions of the coupler have negligible influence on the performance of the device. Hence, the device is wavelength-insensitive and high-dimensional-tolerant. As shown in Figure 10g,h, the coupling and cross-coupling ratios are presented when injecting light into the horizontal and vertical single-mode waveguide, respectively. The mode multiplexer enabled multiplexing of the $LP_{01}$ and $LP_{11}$ modes across a large bandwidth of 400 nm while featuring less than −20 dB crosstalk, high mode extinction ratios exceeding 20 dB and insertion losses well below 2 dB.

#### 4.2.2. Mode Multiplexers Based on Photonic Lantern

**Figure 11:**
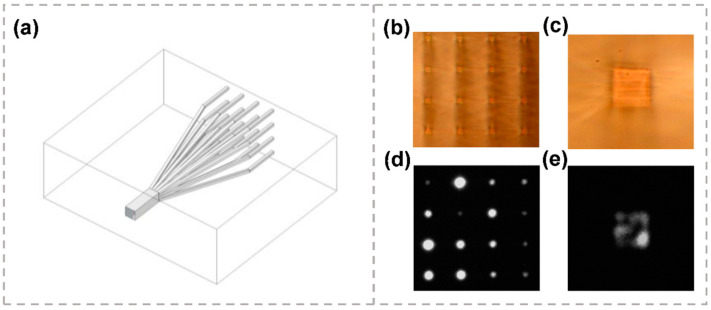
(**a**) Three-dimensional schematic of photonic lantern structure. (**b**) Microscope image of the 4 × 4 single mode array output facet of the photonic lantern. (**c**) Microscope image of the multi-mode waveguide output facet of the photonic lantern. (**d**) Nearfield profiles at the single mode array waveguide output produced by injecting 1550 nm light into the multi-mode inputs. (**d**) Nearfield profiles at the multi-mode waveguide output produced by injecting 1550 nm light into the opposite end [120]. Reproduced from [120] with permission of the Optica Publishing Group, 2022.

A photonic lantern adiabatically merges 16 SMF into a single MMF that supports 16 modes, the 3D schematic of photonic lantern structure is shown in Figure 11a [120]. On one side, 16 single mode waveguides were arranged in a two-dimensional 4 × 4 array with the inter-waveguide spacing of 50 μm. On the other side, these waveguides were brought together to form a single multi-mode waveguide. The microscope images of the 4 × 4 single mode array output facet and the multi-mode waveguide facet are shown in Figure 11b,c, respectively. When the 1550 nm light is injected into the multi-mode inputs, the nearfield profiles at the single mode array waveguide output is shown in Figure 11d. Additionally, when the 1550 nm light is injected into the single mode side input, the nearfield profiles at the multi-mode waveguide output is shown in Figure 11e. The entire device had an average insertion loss of 5.7 dB at 1539 nm. To realize the effective mode-division multiplexing, the signal in each N core will couple to an orthogonal combination of MMF modes when identical SMFs are used [121,122]. A 57-channel mode multiplexer, which consisted of 19 separate three-port photonic lanterns arranged in a hexagonal array, was fabricated by femtosecond laser direct writing [123]. The device would be suitable for the transmission of 114 spatial and polarization modes in a hybrid MCF/FMF fiber with 19 uncoupled three-mode cores. The laser inscription method is also suitable for the fabrication of spatial multiplexers with non-hexagonal core arrangements and where the cores support different numbers of spatial modes within the same fiber. This flexibility could provide additional degrees of freedom for optimizing the design of fibers with multiple few-mode cores. When applying the device, coherent detection and MIMO post-processing are required to recover the transmitted signal because a particular spatial mode in the FMF does not map to a specific single-mode output.

To solve the issue above, mode-selectivity is achieved through introducing asymmetry by using dissimilar single-mode waveguides so that each single-mode input maps to a specific mode at the multimode output [124,125,126]. A femtosecond laser-inscribed six-mode integrated mode-group-selective photonic lantern was demonstrated [127]. The 70 mm long-device consisted of six waveguides. The six single-mode waveguides were arranged at the input in a linear array to match a 127 µm pitch fiber array. After remapping into a pentagon with a central waveguide, the waveguides gradually transited over a 50 mm length to an 8 µm radius pentagon with one waveguide in the center, forming a composite few-mode waveguide.

### 4.3. Fan-In/Fan-Out Devices

Multicore fibers (MCFs) consisting of either a one- or two-dimensional array of guiding cores have been applied in a number of fields including sensing and communications [128,129]. The coupling of light in and out MCFs is a significant problem due to the close proximity and geometrical arrangement of the cores [130]. Femtosecond laser fabrication is capable of creating waveguides that map from an arbitrary 2D arrangement to a linear array. Hence, the femtosecond laser-inscribed fan-out device is of great interest to the MCF field because of its capability to allow the coupling of light in and out MCFs with any core geometry [131,132].

The first femtosecond laser-inscribed fan-out device for the coupling between a 4 × 1 fiber V-groove array (FVA) and a 2 × 2 core array MCF was demonstrated [40]. The fabricated fan-out device consisted of three linear sections connected end-to-end. At each side, a 2.5 mm-long “run-in” section was placed to directly couple with the MCF or FVA cores. The middle section was used to remap the geometric arrangement. At the MCF coupling end of the fan-out, the four run-in sections were arranged in a 50 μm × 50 μm array to match the MCF. At the FVA coupling end of the fan-out device, the four run-in sections were arranged in a one-dimensional array with a 250 μm spacing to match the core spacing of the FVA. The average insertion loss per core of 5.0 dB in the 1.55 μm spectral region was obtained. Furthermore, the group reported a prototype three-dimensional 121-waveguide fan-out device capable of reformatting the output of a 120-core multicore fiber (MCF) into a one-dimensional linear array by the same fabrication method [131]. When used in conjunction with an actual MCF, an overall throughput loss of about 7.0 dB was obtained.

**Figure 12:**
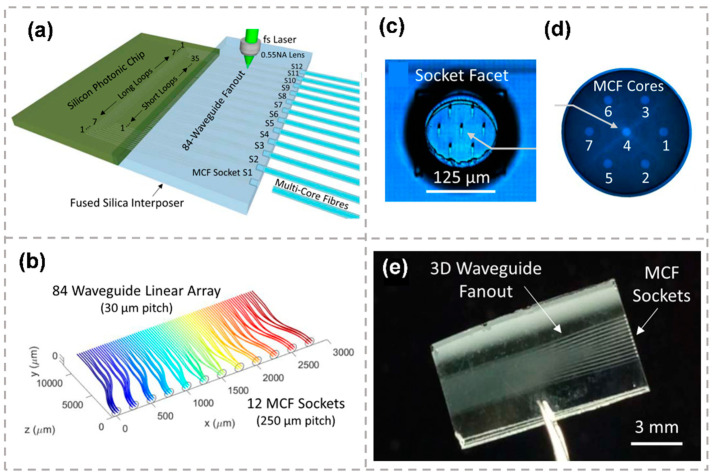
(**a**) A femtosecond (fs) laser inscribes an optical waveguide fanout together with etching tracks that open into MCF alignment sockets after chemical etching. (**b**) Schematic for the waveguide routing design, fanning out from a linear array (SiP chip at back) to 12 socket positions for MCF packaging. (**c**) End view of a MCF socket, showing precisely positioned waveguides on the buried facet. (**d**) Image of a cleaved MCF facet showing hexagonal arrangement of the seven core waveguides and their labels. (**e**) Photo of the fabricated fused silica fan-out device [129]. Reproduced from [129] with permission of the IOP Publishing, 2022.

An 84-channel fan-out device in fused silica was also introduced for high-density edge coupling of multicore fibers to a SiP chip [129]. Figure 12a presents the schematic of the 3D fan-out device. As shown in Figure 12b, the 84 waveguides fan out from a densely packed linear array of 30 um pitch at the SiP chip side into a 2D pattern composed of twelve sockets of seven-core waveguide arrangement matching with the MCF. At the SiP chip side, each silicon waveguide ends with an air-suspended cantilever inverse taper serving as a mode expander for better matching with the 3D fan-out device waveguide mode size. Figure 12c,d show the end facets of the waveguide sockets and MCF, respectively. On the opposing facet of the fan-out device, the twelve sockets were arranged in a linear array of 250 μm pitch, providing ample space for aligning and adhesive dispensing with the 125 μm diameter MMF. Each inscribed waveguide in the fan-out device followed a sequence of straight and circular arc bends, which were optimized to obtain minimum path length difference, low bending loss and crosstalk. The 12.7 mm-long 3D femtosecond laser-inscribed fan-out device was shown in Figure 12e. For the fully packaged photonic system, the insertion losses were measured ranging from −6.9 to −10.9 dB at 1310 nm and from −5.5 dB to −8.2 dB at 1550 nm. The channel crosstalk was better than −20 dB.

## 5. Photonic Wire Bonding

Photonic wire bonding is introduced as a novel concept for automated 3D fabrication of optical chip-to-chip interconnections. Three-dimensional (3D) nano-printing of freeform optical waveguides, also referred to as photonic wire bonding, is based on the femtosecond laser two-photon polymerization (TPP) technology, which is a powerful and potential technology to fabricate true three-dimensional (3D) micro/nanostructures of various materials with sub-diffraction limit resolution. The shape and the trajectory of photonic wire bonds can be adapted to the mode-field profiles and the positions of the chips, which is a key advantage [133,134].

The first single-mode PWB link between two different nanophotonic SOI chips was demonstrated in 2012 [42]. Femtosecond laser two-photon polymerization was deployed to fabricate 3D photonic wire bonds that connect standard SOI waveguides. The shapes of the photonic wire bonds (PWB) were adapted to the actual positions of the integrated waveguide facets, obsoleting the high-precision alignment of optical devices. To precisely attach the wire bond to the SOI waveguides, the lateral positions of the inverse tapers were visually determined by the microscope camera through the lithography objective. To get the accurate vertical position, the focus of the image was optimized manually. The achievable alignment tolerances of the photonic wire bond with respect to the SOI waveguides were estimated to be better than 500 nm in all directions. The high-quality three-dimensional taper can ideally realize adiabatic conversion and single-mode transmission, to realize low insertion loss of 2.5 ± 1.2 dB between 1240 nm and 1580 nm, and with losses of 1.6 ± 0.4 dB in the C-band (1530–1565 nm). The signal with a 5.25 Tbit/s aggregate data rate was transmitted through a photonic wire bond without any measurable signal degradation, which proves the reliability of the photonic wire bond. Photonic wire bonding can play a vital role in interconnects with spatial densities in the Pbit/s/mm range.

Photonic wire bonding can also connect multi-core fibers and single-mode SOI waveguides with low insertion loss. In the demonstration, the photonic wire bond had tapered sections on the fiber enfaces as well as towards the SOI chip for adapting the mode fields. Between the tapered structures, the PWB had a round cross-section, the axis of which follows a 3D trajectory in space. The PWB trajectory was carefully designed and optimized to minimize the transmission loss. The technique does not need to consider the strict coupling alignment conditions between devices, greatly simplifies the coupling process, and can realize full-automatic coupling. The insertion losses between the multi-core fibers and single-mode SOI waveguides were down to 1.7 dB, which has much potential for further improvement [135].

A flexible fabrication process enables photonic wire bonding to match various device platforms. A photonic wire bonding connecting InP-based horizontal-cavity surface-emitting lasers (HCSEL) to passive silicon photonic circuits with insertion losses down to 0.4 dB was demonstrated [136]. To minimize optical loss, the design of a PWB should obey several constraints. At first, the end facets of the PWB must overlap with the connecting waveguides on both sides. Secondly, the starting and ending orientation of the PWB axes must coincide with the connecting waveguide axes. Thirdly, the trajectory of the PWB axis is to be chosen such that intersections with obstacles such as fibers, chip edges or other PWB are avoided. Finally, increased losses by a strong curvature of the trajectory and a large length should be avoided, and a suitable compromise should be found. Hence, hybrid photonic multi-chip assemblies that combine known good devices of different materials together to form high-performance hybrid multi-chip modules can be achieved by photonic wire bonding.

**Figure 13:**
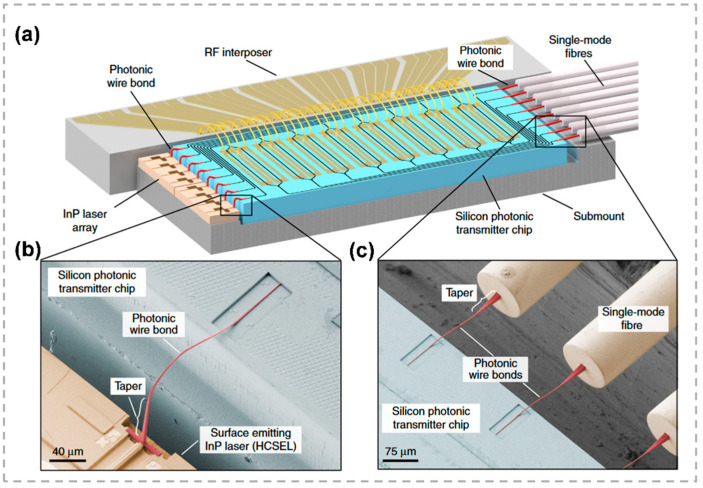
(**a**) Concept and implementation of hybrid multi-chip modules by 3D nano-printing of photonic wire bonds. (**b**) Interface between an InP laser chip and the silicon photonic transmitter chip. (**c**) Fiber-to-chip interface [137]. Reproduced from [137] with permission of the Springer Nature, 2022.

Furthermore, the photonic wire bonds which connect arrays of silicon photonic modulators to InP lasers and single-mode fibers was also demonstrated. Figure 13a shows the concept and implementation of hybrid multi-chip modules by 3D nano-printing of PWBs. Figure 13b displays the interface between an InP laser chip and the silicon photonic transmitter chip. The light source was realized as a HCSEL, which consisted of a waveguide-based optical cavity in the substrate plane and an etched 45° mirror that redirects the light towards the substrate-normal direction, while Figure 13c displays the fiber-to-chip interface. Since the waveguide shape and position can be freely set in three-dimensional space, highly integrated fan-in and fan-out connecting based on photonic wire bonding can be realized. The insertion losses of the photonic wire bonds are measured to be (0.7 ± 0.15) dB. The resilience of photonic wire bonds in environmental stability and at high optical power were tested to be of good performance [137].

It is worth mentioning that the PWB prototypes exhibit excellent mechanical and chemical stability: Repeated testing of the waveguides over several weeks with optical powers of up to 100 mW did not reveal any degradation of the transmission loss. The mechanical stability of the structures is excellent because that they are not affected by manual handling of the chip with tweezers or by intensive rinsing in water after the development step. Moreover, the fabricated wire bonds exhibit strong adhesion to the silicon chip surface and do not detach even when treated with oxygen plasma or when immersed in acetone [42].

## 6. Conclusions

In summary, we focus on femtosecond laser-fabricated photonic chips for optical communications in this review article. We first briefly introduce the basic concept of femtosecond laser fabrication and different types of laser-inscribed waveguides. Then, the on-chip laser-fabricated active devices and passive devices are presented. By direct fabrication and flexible 3D configuration of waveguide-based optical components in a wide spectrum of optical materials, various optical functionalities including laser generation, laser amplification, EO modulation, frequency conversion, polarization–division multiplexing, mode–division multiplexing, multi-core multiplexing and photonic wire bonding have been achieved. The devices with these functionalities can be easily integrated with novel optical materials for optical communication applications.

**Figure 14:**
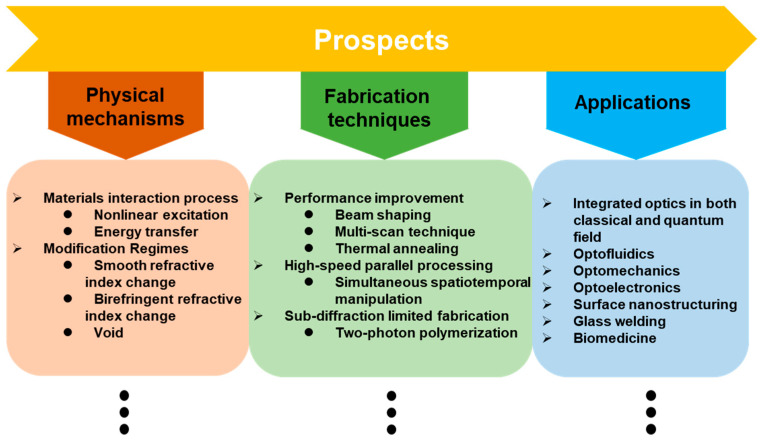
Prospects for femtosecond laser-fabricated photonic chips.

The femtosecond laser fabrication technology has shown the powerful ability and unique capability to construct diverse waveguide devices with high qualities for future photonic networks. As shown in Figure 14, the future efforts in this research would mostly focus on three directions: exploring the undisclosed physical mechanisms, developing new fabrication techniques, and extending the application range. It is well known that femtosecond laser fabrication provides unique advantages such as the suppression of thermal diffusion, nonlinear multiphoton excitation of carriers, deterministic optical breakdown threshold, internal modification of transparent materials and reproducible nanoscale resolution. However, the physical mechanisms of material modification are not yet fully understood, especially after the free-electron plasma transferring its energy to the lattice [138]. Understanding these physical mechanisms requires the detailed diagnosis of ultrafast plasma dynamics inside transparent materials, which would enable us to make better use of this technology. The rich variety of parameters provided by femtosecond laser pulses has allowed femtosecond laser beam shaping to be extensively investigated. Early efforts were mainly focused on spatial or temporal shaping of ultrafast laser pulses to improve waveguide quality. Several beam-shaping technologies have been proposed, including slit beam shaping, astigmatic beam shaping and spatial light modulator (SLM) beam shaping [139,140,141,142,143,144]. Multi-scan technology and thermal annealing technologies are applied to improve the quality of the inscribed waveguide. Beam shaping effects also aimed at high-speed parallel processing and sub-diffraction limited fabrication, such as simultaneous spatiotemporal manipulation and two-photon polymerization technology [145]. The research of various materials would promote the development of applications based on femtosecond laser fabrication. The femtosecond laser-fabricated chips based on glass and crystals have also been applied to integrated optics and photonics in both classical and quantum fields. Surface nano-structuring can be applied not only on the surfaces of various materials including metals, glass, ceramics, semiconductors, and insulators, but also advanced materials such as graphene. Several laser-fabricated graphene devices, such as flexible electrodes, photovoltaic devices, sensors, and micro-supercapacitors, have been demonstrated [26]. The fabrication of 3D polymer micro- and nanocomponents by TPP, such as optofluidics, optomechanics, glass welding and biomedicine becomes more and more open for not only scientific research in various fields, but also for product development [44,146,147].

The wide range of material platforms, diverse fabrication technologies and large quantities of application demands indicate the great potential of the femtosecond laser fabrication technology. It is reasonable to expect further exploring emerging/potential photonic functionalities and systems in the near future.

## Data Availability

Not applicable.
